# The *Drosophila mojavensis Bari3* transposon: distribution and functional characterization

**DOI:** 10.1186/1759-8753-5-21

**Published:** 2014-07-08

**Authors:** Antonio Palazzo, Roberta Moschetti, Ruggiero Caizzi, René Massimiliano Marsano

**Affiliations:** 1 Dipartimento di Biologia, Università degli Studi di Bari “Aldo Moro”, Via Orabona 4, 70125 Bari, Italy

**Keywords:** Transposon, Transposase, *Tc1*-like elements, *Bari3*, *Drosophila mojavensis*, Transposase transcript splicing, Nuclear localization signal, Luciferase promoter assay

## Abstract

**Background:**

*Bari*-like transposons belong to the *Tc1-mariner* superfamily, and they have been identified in several genomes of the *Drosophila* genus. This transposon’s family has been used as paradigm to investigate the complex dynamics underlying the persistence and structural evolution of transposable elements (TEs) within a genome. Three structural *Bari* variants have been identified so far and can be distinguished based on the organization of their terminal inverted repeats. *Bari3* is the last discovered member of this family identified in *Drosophila mojavensis*, a recently emerged species of the Repleta group of the genus *Drosophila*.

**Results:**

We studied the insertion pattern of *Bari3* in different *D. mojavensis* populations and found evidence of recent transposition activity. Analysis of the transposase domains unveiled the presence of a functional nuclear localization signal, as well as a functional binding domain. Using luciferase-based assays, we investigated the promoter activity of *Bari3* as well as the interaction of its transposase with its left terminus. The results suggest that *Bari3* is transposition-competent. Finally we demonstrated transposase transcript processing when the transposase gene is overexpressed *in vivo* and *in vitro*.

**Conclusions:**

*Bari3* displays very similar structural and functional features with its close relative, *Bari1*. Our results strongly suggest that *Bari3* is an independent element that has generated genomic diversity in *D. mojavensis*. It can autonomously transcribe its transposase gene, which in turn can localize in the nucleus and bind the terminal inverted repeats of the transposon. Nevertheless, the identification of an unpredicted spliced form of the *Bari3* transposase transcript allows us to hypothesize a control mechanism of its mobility based on mRNA processing. These results will aid the studies on the *Bari* family of transposons, which is intriguing for its widespread diffusion in Drosophilids coupled with a structural diversity generated during the evolution of *Bari*-like elements in their host genomes.

## Background

A consistent fraction of eukaryotic genomes is composed of transposable elements (TEs). Although they were originally considered as ‘selfish’ or ‘junk’ elements [1,2] and as potentially representing endogenous mutagens, they are now believed to represent one of the major forces driving the evolution of genes and genomes [3-5].

DNA-based TEs belong to the Class II of transposons and use a DNA-mediated mode of transposition and self-encoded transposases to catalyze the transposition reaction, unlike Class I elements that move via reverse transcription of RNA intermediates. Seventeen cut-and-paste DNA transposons superfamilies have been discovered so far [6], with the best studied undoubtedly being the *Tc1-mariner* superfamily.

The *IS630-Tc1-mariner* (or ItmDx(D/E superfamily)) [7] constitutes the largest group of cut-and-paste Class II transposons. These elements are up to 2 Kbp in length and usually contain a single transposase-encoding gene, typically flanked by two short terminal inverted repeats (TIRs). The transposase of these elements is sufficient to catalyze the transposition reaction *in vitro*[8] by recognition of the TIRs, explaining in part the wide phylogenetic occurrence of *Tc1/mariner-*like elements [9].

The complex dynamics underlying the invasion and the persistence of TEs in a genome could be better understood by studying different elements belonging to the same family and hosted in genomes of different species [10]. Furthermore this kind of approach could give clues in improving the transposition efficiency of TEs in order to establish new transposon-based integration tools [11]. As an example, the *mos1* element discovered in *Drosophila mauritiana* has been used as starting point to isolate the *Himar1* element in the horn fly *Haematobia irritans,* which transposition efficiency has been further improved *in vitro*[8,12].

In this view the *Bari* family potentially represents an interesting case study in the *Drosophila* genus.

Three related *Bari* sub-families (*Bari1*, *Bari2* and *Bari3*), differing in their structural organization and their potential transposition ability, are known to exist in different *Drosophila* species [13,14]. While elements related to *Bari1* and *Bari3* can be either potentially autonomous or not, elements related to *Bari2* are all non-autonomous [13,14]. *Bari*-like elements belong to the IR-DR group of the *Tc1* lineage, comprising elements with terminal ends of about 250 bp in length. This group also includes other *Drosophila*-related TEs such as *S*[15], *Minos*[16], and *Paris*[17], as well as non-insect members like the *Sleeping Beauty (SB)*[11] and the *Frog Prince (FP)*[18] transposons, reconstructed from fish and amphibian genomes, respectively. These elements encode transposases containing a predicted functional bipartite nuclear localization signal (NLS), two helix-turn-helix (HTH) motifs in the N-terminal region and an acidic DD34E triad in the C-terminal region [19-21].

Most of the information on the *Bari*-like elements is related to the *Bari1* element probably due to its presence into the *D. melanogaster* genome in a putatively active form, as demonstrated by direct [22] and indirect [23] evidence.

Recently the NLS and the DNA binding site of the transposase encoded by the *Bari1* element have been functionally characterized [24]. The TIRs of *Tc1-mariner* elements possess two or three direct repeats (DRs), that are the putative binding sites for the transposase and are necessary for the transposition of autonomous elements [21,25,26]. *Bari1* has three DRs in its terminal sequences that are all bounded, although with different efficiency, by the *Bari1* transposase [24].

*Bari3* is the last discovered member of the *Bari* family. It has been identified in the genome of the emerging species *D. mojavensis,* but homologous sequences can be also identified in the sequenced genomes of the phylogenetically distant species *D. pseudoobscura*, *D. persimilis and D. willistoni*[14]. Its structural characteristics, that is, long TIRs with three DRs bracketing a transposase coding region, allowed the determination of the evolutionary dynamics acting on the transposon termini [14]. Furthermore, at least ten identical copies of this element can be detected in the sequenced genome *D. mojavensis*, suggesting its very recent transposition activity. Previous studies concerning the phylogenetic distribution of the *Bari*-like elements have disclosed inconsistencies with the species phylogeny that have demonstrated [27] or postulated [14] ancient horizontal gene transfer events.

These observations along with our previous functional study of the *Bari1* transposon [24], prompted us to investigate and compare this new member of the *Bari* family in order to gain insight into the biology of this transposon family.

Here, we show that *Bari3* is a widely distributed transposon in the *D. mojavensis* populations with a variable copy number within the genome of different subspecies. Similarly to *Bari1*, the *Bari3* transposase is able to bind the TIRs of the transposon and localizes in the nucleus of *Drosophila* and human cells. We have also investigated the internal promoter of *Bari3* and the transposon-transposase interaction. Furthermore, transient transposase gene overexpression allowed the isolation of an unexpected spliced transcript in cultured cells and in embryos. These data are discussed in the light of previous studies concerning a putative transposition control of the *Bari* family.

## Results

### The distribution of *Bari3* in the genome of *Drosophila mojavensis*

We previously reported, using *in silico* approaches, the recent invasion of the transposon *Bari3* in the genome of the emerging *Drosophila* species, *D. mojavensis,*[14]. *D. mojavensis* is endemic to the Sonoran Desert of North America, with different subpopulations specialized in feeding on different necrotic cactus tissues and showing both genetic differentiation and reproductive isolation [28-30].

**Figure 1:**
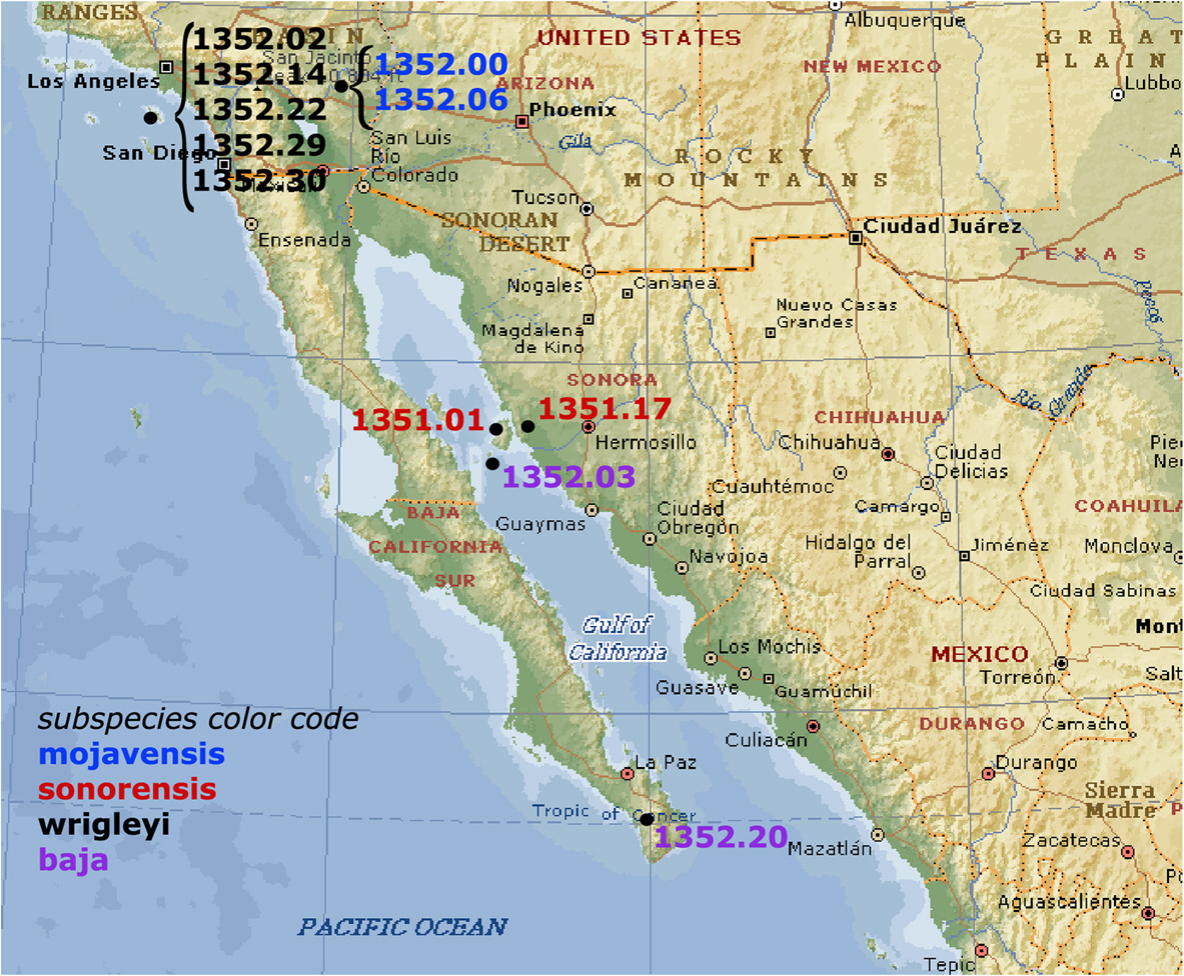
**Geographical origin of the *Drosophila mojavensis *strains analyzed in this study.** The prefix 15081 has been omitted for space restriction (see Table 1). *D. mojavensis* subspecies are indicated according to the color code showed.

In order to estimate the activity of the *Bari3*, we analyzed its distribution in the population of *D. mojavensis* collected in different geographical regions of California and Mexico (Figure 1 and Table 1).

**Table 1:** ***Drosophila mojavensis *** **strains used in this study**

**DSSC code**	**Subspecies**^**a **^**(race**^**b**^**)**	**Collection place**^**a**^	**Collection date**^**a**^	***Bari3 *** **Southern/FISH signals detected**^**c**^
**15081-1352.00**	mojavensis (A)	Chocolate Mountains, Riverside County, California	N/D	1
**15081-1352.06**	mojavensis (A)	Chocolate Mountains, Riverside County, California	N/D	½
**15081-1351.01**	sonorensis (BI)	Tiburon Island, Gulf of California Mexico	(1964)	1 (faint)
**15081-1351.17**	sonorensis (BI)	Punta Onah Sonora, Mexico	(1988)	2
**15081-1352.02**	wrigleyi (C)	USC marine station, Catalina Island, California	(1991)	9/9
**15081-1352.14**	wrigleyi (C)	Santa Catalina Island, California	(2002)	11/16
**15081-1352.22**	wrigleyi (C)	Catalina Island, California	(2002)	10
**15081-1352.29**	wrigleyi (C)	Little Harbor, Catalina Island, California	(2004)	9
**15081-1352.30**	wrigleyi (C)	Catalina Island, California	(2002)	5
**15081-1352.03**	baja (BII)	San Esteban Island Gulf of California Mexico	(1965)	ND/5
**15081-1352.20**	baja (BII)	Cape Region, Santiago, Baja California South Mexico	(1996)	5/7

^a^Data from Drosophila Species Stock Center (DSSC).

^b^Race definition is according to Pfeiler *et al*. [31].

^c^This study.

N.D., not determined.

A full length *Bari3* element was cloned from the genome of the sequenced *D. mojavensis* strain (pT/moja11) using a PCR-based strategy (see Methods section) [32]. Sequence and structure of this element are described in Additional file 1.

**Figure 2:**
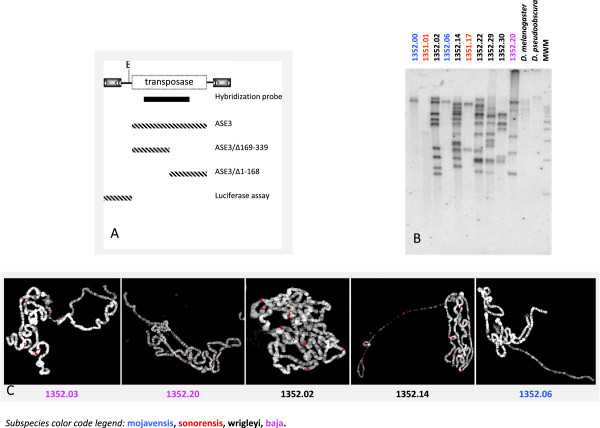
***Bari3 *distribution in the genome of *Drosophila mojavensis*. A)** Schematic representation of the *Bari3* transposon. The EcoRI site used for the genomic analyses and the position of the probe (black bar) are showed. Dashed bars represent the transposon fragments tested in this work. **B)** Southern blot hybridization of DNA samples extracted from ten *D. mojavensis* populations MWM, 1Kb DNA molecular weight marker (Promega). **C)** Fluorescence In Situ Hybridization (FISH) on polytene chromosomes prepared from five *D. mojavensis* strains. Merged images (DAPI and Cy3) are shown. Hybridization signals are pseudo-colored in red. The subspecies color code legend reported in the bottom of the figure refers to the hybridization experiments.

The DNA extracted *en masse* from ten *D. mojavensis* populations was digested with the endonuclease EcoRI and analyzed by Southern blot hybridization. We used an internal 592-bp fragment (Figure 2A) as a probe, subcloned from the full-length *Bari3* element. To avoid nonspecific detection of divergent sequences related to transposon relics, we applied high-stringency conditions for our hybridization experiments. The pattern obtained is shown in Figure 2 (panel B) and clearly indicates variability in both the copy number and genomic distribution of the *Bari3* elements among the populations analyzed. We estimate that the *baja* and *wrigleyi* subspecies contain from 5 to 11 copies of the transposon, while the *mojavensis* and *sonorensis* subspecies contain 1 to 3 copies of *Bari3*. As expected, only very faint bands can be detected in the distant species *D. melanogaster* and *D. pseudoobscura*, confirming that the *Bari3* element of *D. mojavensis* is quite divergent from the *Bari3* element of *D. pseudoobscura* and from the *Bari1* and *Bari2* elements of *D. melanogaster*[14].

We used the sequence of *Bari3* as our query, to perform a BLAST analysis against the WGS database of *D. mojavensis*. These experiments revealed ten full-length copies of *Bari3* and at least ten defective ones, slightly divergent in sequence and bearing mostly terminal truncations. This result, summarized in Additional file 2, is in line with the hybridization pattern observed for the 1352.22 strain and suggests that at least part of the differences observed are due to degenerated *Bari3* copies.

We further characterized the *Bari3* insertion sites within the genome of *D. mojavensis* populations by analyzing the *in situ* hybridization pattern over polytene chromosomes of five different strains. As shown in Figure 2 (panel C), a variable number of hybridization sites were revealed that are substantially in accord with the number of polymorphic bands seen in Southern hybridization experiments. Taken together, these results strongly indicate a recent transposition activity of *Bari3*.

### Analysis of the *Bari3* transposase domains

To gain further insight into the *Bari3* transposon, we started a preliminary characterization of its transposase. Typically, the NLS is present at the N-terminus of the transposase in *Tc1-mariner* elements, although other elements may present the NLS at the C-terminus. The presence of a functional nuclear import domain was firstly assayed because it represents a necessary condition for the mobility of a transposon.

Immuno-detection was performed in cells transiently overexpressing a V5-His tagged *Bari3* transposase. Subcellular localization was assayed in two model cellular systems, the *Drosophila* S2R + and the human HepG2 cells. With the aim to localize the NLS domain within the transposase protein we tested either the full-length (ASE3) or truncated versions (ASE3/Δ169-339 and ASE3/Δ1-168) of the transposase fused to the V5-His tag in the above mentioned cell types. A schematic representation of the transposase gene fragments tested in these experiments is shown in Figure 2A. The cellular localization of the expressed proteins was then visualized by using a monoclonal anti-V5 antibody.

**Figure 3:**
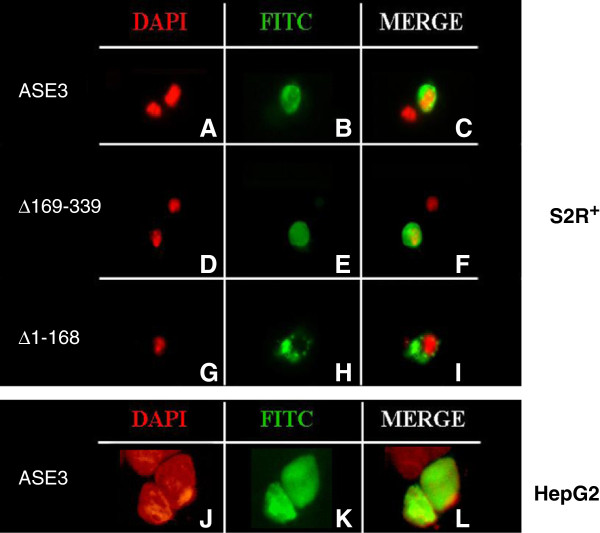
**Subcellular localization of the *Bari3 *transposase.** Upper Panel. Localization of the full-length (ASE3), the N-terminal (Δ169-339) and the carboxyl terminal (Δ1-168) portion of the *Bari3* transposase in S2R + cells. Lower Panel. Localization of the full-length *Bari3* transposase in HepG2 cells. With 4',6-diamidino-2-phenylindole (DAPI) signal **(A, D, G, J)**; Fluorescein isothiocyanate (FITC) signal **(B, E, H, K)**; merged signals **(C, F, I, L)**.

The results are showed in Figure 3. Full-length *Bari3* transposase localizes to the nucleus in both cell types (Figure 3-C and 3-L), indicating that a nuclear import signal is contained within the protein and is functional in both insect and mammalian cells. Furthermore, we mapped the NLS signal within the N-terminal half portion of the protein since a deleted C-terminal construct (Δ169-339) retains its nuclear localization (Figure 3, panel F), while the deleted N-terminal part of the transposase (Δ1-168) does not (Figure 3, panel I).

The presence of additional canonical motifs in the *Bari3* transposase also has been investigated using a combination of *in silico* methods. The primary sequence of the *Bari3* transposase was compared to other functional *Tc1-mariner* like transposase sequences including *SB*, *FP*, *minos*, *Hsmar* and *mos1* and the recently characterized *Bari1* in a multialignment.

**Figure 4:**
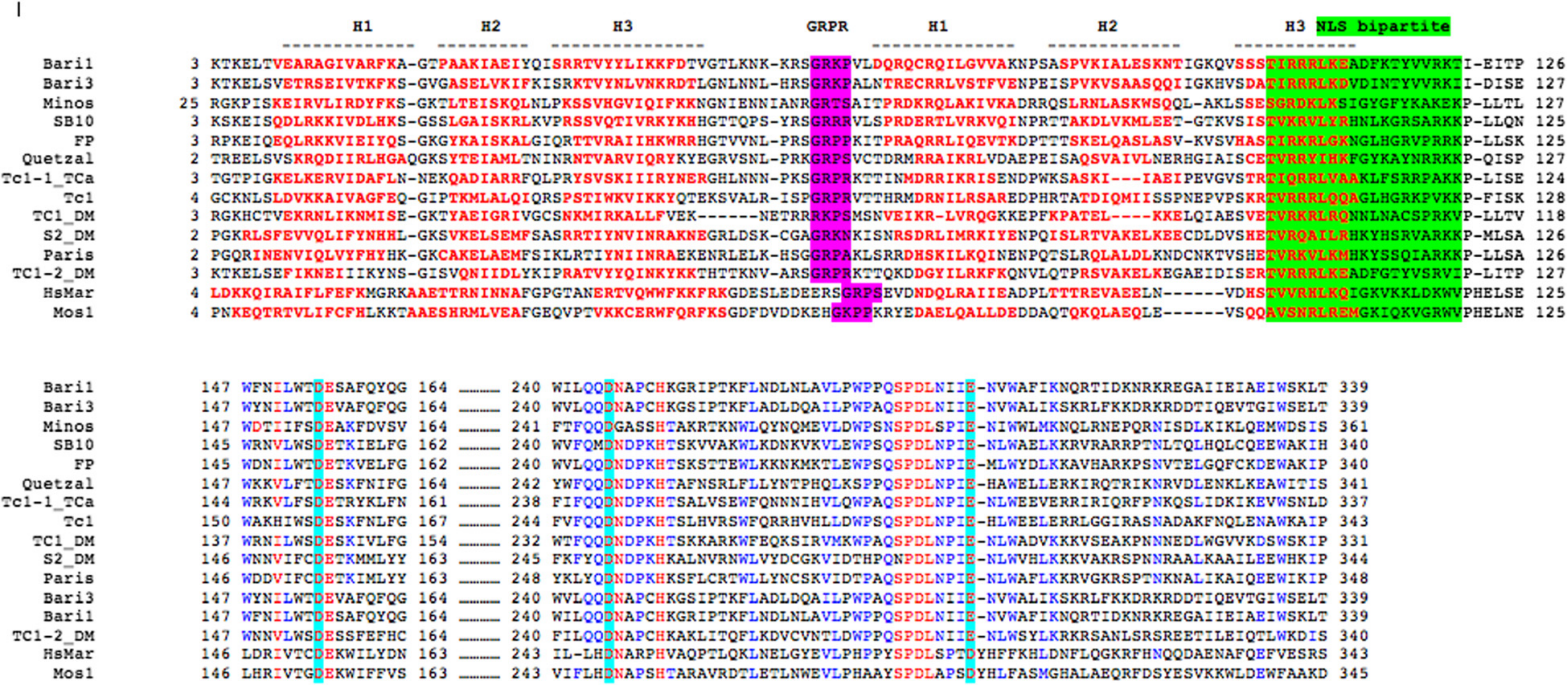
**Multiple alignment of *Tc1-mariner *transposases.** Residues of the DNA binding domain (consisting of the H1-H3 alpha helices and indicated above the alignment) are red boldfaced, the GRPR domain is highlighted in purple, nuclear localization signal (NLS) is highlighted in green and the acidic triad of the catalytic domains (DDE) is highlighted in turquoise.

The identification of the HTH structure of the analyzed transposase was performed by *in silico* prediction with PredictProtein [33] and the predicted alpha helices were annotated on a multiple alignment generated with Multalin [34] (Figure 4).

A bipartite DNA binding domain thought to be responsible for recognition of the transposon termini can be easily detected at the N-terminus of the protein. This domain is divergent in sequences among the compared transposases, but the predicted alpha helices of both HTH motifs occupy a similar position with respect to each other, suggesting the functional conservation of these divergent sequences. As demonstrated for other *Tc1*-like elements, the N-terminal domain of the transposase may also contain motifs mediating dimerization (or tetramerization) of the transposase [21].

A GRPR-like motif (GRKP) motif characteristic of the homeo-domain proteins [35] is also present at position 59 of *Bari3* and between the two HTH motifs. This domain precedes an additional HTH region (that is, the homeo-like domain) in all the transposases aligned. The multiple alignment also highlights the presence of a putative bipartite NLS rich in basic amino acids, whose functionality has been experimentally demonstrated for *Bari3* transposase (see Results above and Figure 3). Finally, the catalytic domain, characterized by the typical DDE motif, is also recognizable in the primary sequence of *Bari3*.

### Overexpression of *Bari3* transposase produces spliced transcripts

We recently reported that *Bari1* transcripts can be subjected to post-transcriptional processing under specific experimental conditions [24]. The *Bari1* processed transcripts could be theoretically involved in the regulation of the transposition as they potentially encode for truncated transposase molecules, which can poison the active transposon-transposase complex [24].

We have investigated the possibility that *Bari3* could also generate similar processed transcripts.

**Figure 5:**
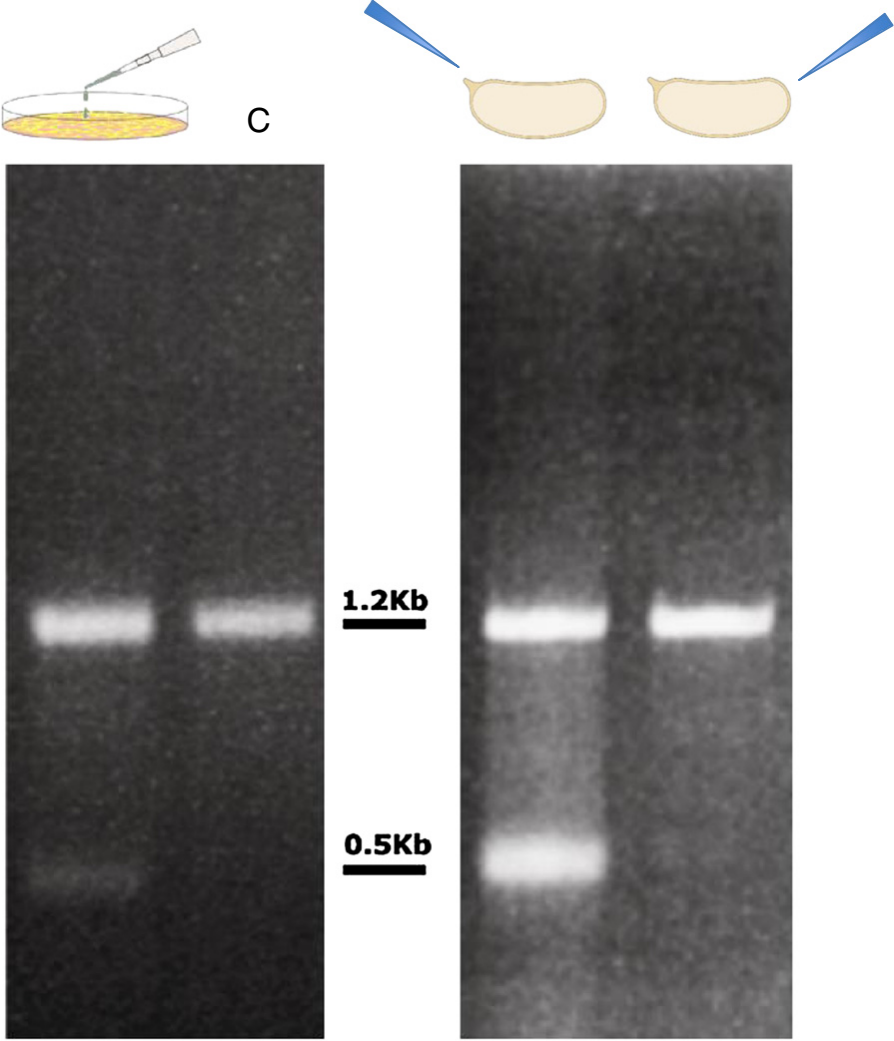
**Reverse-transcription polymerase chain reaction (RT-PCR) results.** Left panel, RT-PCR results from transfected cells. C = control indicating the expected full-length transcript. Right panel, RT-PCR results from embryos injected in the anterior (left-most lane) or in the posterior (right-most lane) pole. Position of bands relative to the 1Kb DNA Ladder (New England Biolabs) is indicated.

RT-PCR experiments performed after transient overexpression of *Bari3* transposase (pAC/ASE3 plasmid) in S2R + cells led to the identification of a transcript of unexpected size in addition to the expected full-length transcript (Figure 5 left panel). Sequence comparison of the cloned short cDNA with the full-length transcript sequence reveals a deletion of 699 bp bracketed by canonical GT-AG consensus of the splicing sites (see Additional file 3, panel A). Interestingly, the short cDNA still displays an ORF encoding the last 98 amino acids of the wild type *Bari3* transposase (see Additional file 3, panel B). Therefore, a canonical splicing event is likely to generate an uncommon short transcript of *Bari3* upon overexpression in S2R + cells.

With the aim to confirm this result *in vivo,* we have transiently overexpressed *Bari3* in *D. melanogaster* wild type embryos. We performed two parallel sets of experiments in which embryos were microinjected with the pAC/ASE3 plasmid either in the posterior pole or in the anterior pole. We reasoned that this strategy could give us the chance to analyze the transposase expression in two very different cellular environments of the embryo. Somatic cells reside in the anterior part of the embryo whereas the posterior part is enriched in precursors of germinal cells, that is, the pole cells.

Two transcripts differing in size were detected upon transient overexpression of *Bari3* in the anterior pole of *D. melanogaster* wild-type embryos (Figure 5 right panel). The pattern obtained looks identical to the pattern observed in cultured cell experiments. Interestingly, only embryos injected in their anterior pole produced the additional short transcript, while in embryos injected in the posterior pole only a single band, corresponding in size to the expected full-length *Bari3* transcript, is detectable. Sequence comparison of the two short cDNA cloned respectively from transfected S2R + cells and from embryos reveals that they are 100% identical and harbor the same spliced fragment.

### *Bari3* transposon harbors an endogenous promoter and interacts with the transposase

**Figure 6:**
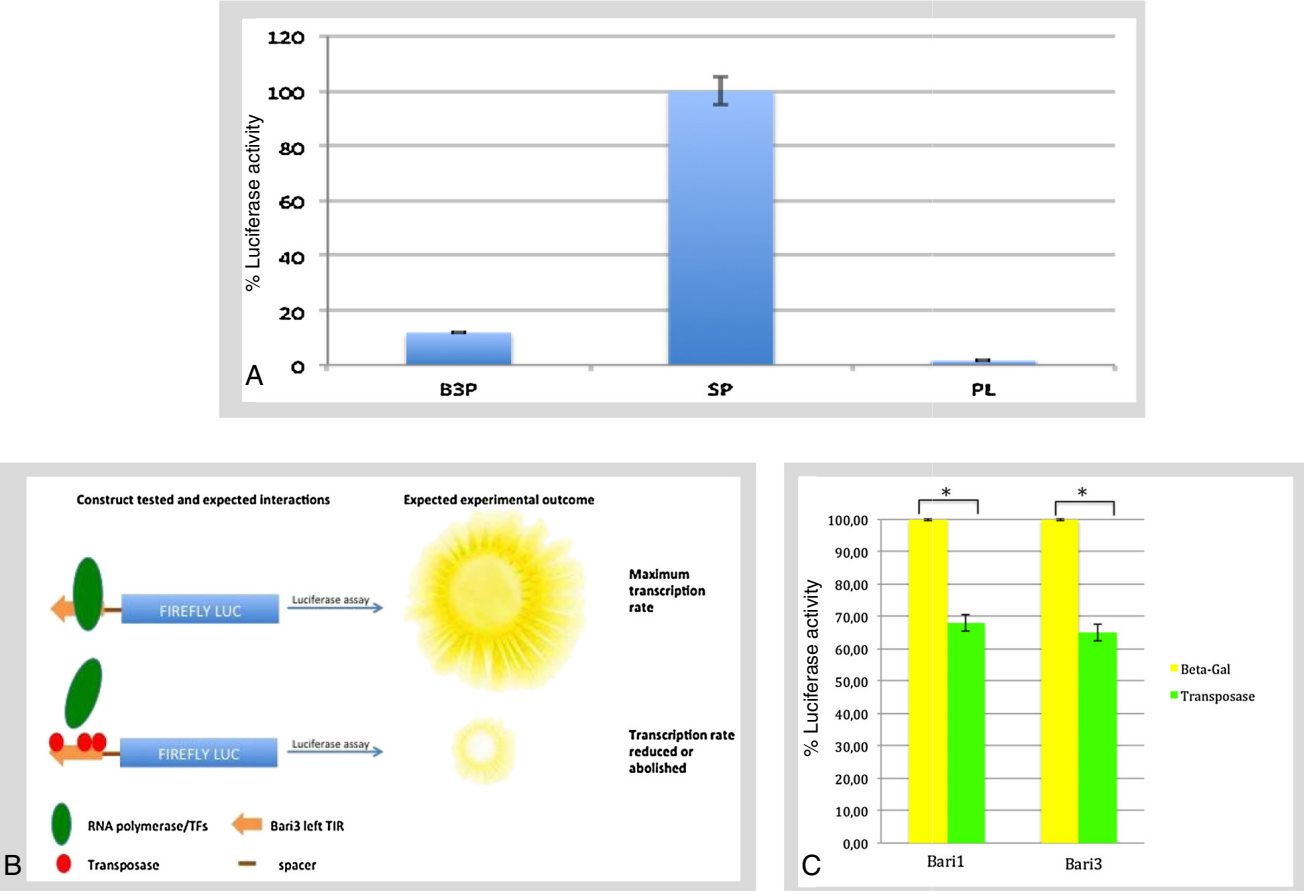
**Luciferase promoter assay and the transposon-transposase interaction. A)** Luciferase promoter assay. B3P, *Bari3* promoter; SP, strong promoter (*copia* promoter); PL, promoter-less. **B**: Rationale of the luciferase activity suppression assay (see main manuscript text for additional details). **C**: Luciferase activity suppression assay results. Asterisks denote *P* <0.05.

*Tc1-mariner* transposable elements usually contain a single gene encoding transposase. To ensure their mobility they need to autonomously drive transcription, and therefore must contain a promoter element in their left (5’) terminus. We have tested the promoter activity of a 356-bp *Bari3* fragment (-1 to -356 relative to the translational start site) using a luciferase assay. The tested fragment overlapping the entire 256-bp left TIR of *Bari3* (plus the 99 bp long spacer, [see Additional file 1]) was directionally cloned into the pGL3B vector, obtaining the pGL3B-Ba3LTIR plasmid. The plasmid was transiently transfected in S2R + cells and the luciferase activity measured. The values obtained were then compared to the values obtained after transfection of the ‘empty’ pGL3B vector (that is, carrying a promoter-less luciferase gene) and to the luciferase activity in cell transfected with a plasmid carrying the strong promoter of the transposable element *copia*[36]. The results shown in Figure 6A suggest that the sequence tested has a detectable promoter activity, roughly 15% with respect to the *copia* promoter. The promoter activity of *Bari3* is also detectable in HeLa cells (data not shown).

We have developed a simple assay based on the luciferase transcriptional suppression to detect the transposase-transposon interaction. The rationale of this procedure is depicted in Figure 6B. Briefly, since the TIR sequence of *Bari3* harbors the transposase binding sites [14] that can overlap the promoter region, we hypothesized that the promoter activity could be negatively affected, totally or at least in part, if *Bari3* transposase is expressed in the same cell, thus disturbing the interaction between transcription factors and their binding sites. The advantages of this method with respect to well-established procedures for *in vitro* (EMSA, CHIP) or *in vivo* (One Hybrid) studies, already used in the characterization of the TIR sequence of *Bari1*[24], are the low costs and fast experiments. We performed this test in HeLa cells due to their greater tractability in terms of transfection efficiency and growth respect to S2R + cells, and because we observed *Bari3* promoter activity also in this experimental system (not shown).

In order to validate this procedure we used the previously validated interaction of *Bari1* left TIR and the *Bari1* transposase as a positive control [24].

HeLa cells were transfected with the pcDNA/ASE3 plasmid expressing the *Bari3* transposase. Then, they were further transfected with the pGL3B-Ba3LTIR plasmid 8 hours after the first transfection. The luciferase activity was measured after 24 hours and compared to the luciferase activity measured in cells transfected with the pGL3B-Ba3LTIR plasmid alone. Assuming that cells transfected with pGL3B-Ba3LTIR, in the absence of transposase protein, represent the 100% level of luciferase expression, any significant decrease in luciferase activity can be ascribed to the presence of transposase binding to the DRs on the *Bari3* left TIR. As a negative control, we measured the luciferase activity in cells transfected with a β-Galactosidase-expressing plasmid the protein (in place of the pcDNA/ASE3 plasmid), and then further transfected with the pGL3B-Ba3LTIR plasmid, as described above. The results show a significant lowering of the luciferase activity in cells overexpressing *Bari1* transposase or *Bari3* transposase if compared to the luciferase activity measured in the presence of β-Galactosidase expressed in the same conditions (Figure 6C). Taken together the results obtained indicate that the reduced promoter activity observed can be ascribed to the transposase interaction with the *Bari3* terminal inverted repeat, probably at the DR sites.

## Discussion

The post-genomic era allows identification of novel transposable elements, which can be ascribed to known or new families of the major TEs clades. Besides understanding the potential impact of TEs in genome plasticity, the increasing knowledge on TE biology has found applications both in biotechnology and medicine [37,38]. A growing number of TE-based integration tools have been developed in the past 30 years either starting from reconstructed elements [11] or from intact elements isolated from the more diverse organisms [39,40]. New genomic sequences are promising sources of novel transposons, and their functional characterization would give hints for their use in genetics and biotechnology.

Emerging species are probably a mine of information concerning TEs. The reorganization, repositioning and acquisition of novel TEs by genomes are considered as one of the main pulses in speciation [3,41]. *Bari3* might represent one such case, as it has been isolated in the genome of *D. mojavensis*, a recently diverged species of the Repleta group [42]. *Bari3* has novel structural features compared to other members of the *Bari* families, *Bari1* and *Bari2. Bari1* has imperfect short TIRs bracketing the transposase gene [43], while *Bari2* has identical long TIRs but mutated transposase [13]. Contrary to older elements, such as *Bari1*, that lost TIRs identity but retained transposition activity [22], or such as *Bari2* which accumulated deleterious mutations that impaired its transposition activity, the *Bari3* element present in *D. mojavensis* appears to be a ‘young’ *Bari*-like element possessing a transposase coding region and perfect long TIRs.

While the diffusion of *Bari*-like elements through a wide range of *Drosophila* strains is intriguing, the functional and structural features underpinning the success of these elements to colonize different species remain unknown. Here we focus on four informative aspects of this process, that is, 1) the genomic distribution of *Bari3* across different *D. mojavensis* populations; 2) the presence of an internal promoter able to drive the transcription of the transposase gene; 3) the cellular localization of the transposase and its physical interaction with the transposon; 4) the existence of a post-transcriptional regulation mechanism based on alternative splicing in the control of the transposition of the *Bari3* element.

### The genomic distribution across different *Drosophila mojavensis* populations suggests that *Bari3* is an active element

Based on molecular, morphological and ethological data, which support the differentiation across the geographical distribution of the species, *D. mojavensis* consists of four recognized races. Albeit the limited sample size, our results reflect the genetic variability of different populations of *D. mojavensis* observed in previous population studies [44]. We found that the copy number of *Bari3* is related to the *D. mojavensis* subspecies, and to the distinct geographic region they occupy. For instance the subspecies *mojavensis* (breeding in barrel cactus in Mojave Desert and the Grand Canyon) and *sonorensis* (breeding in organ pipe cactus in Sonora and Southern Arizona) contain few *Bari3* copies. On the contrary the *baja* (breeding in agria in Baja) and *wrigleyi* (breeding in prickly pear in Santa Catalina) subspecies (Table 1 and Figure 2) are characterized by a higher number of insertions. These evidences, taken together, could suggest that environmental factors might have a role in the determination of strain-specific copy number [45].

*In silico* analyses performed in the sequenced strain of *D. mojavensis* (15081-1352.22) identified multiple identical *Bari3* copies, [14] [see Additional file 2], as well as several terminally truncated *Bari3* copies, that may have originated by repair of DNA-breaks induced during transposition [46]; both types of elements are compatible with recent activity of *Bari3*.

### *Bari3* harbors an internal promoter and encodes a putatively active transposase

Transposons need to express their own transposase in order to move within the genome. We have demonstrated that the sequence upstream the translational start site of *Bari3* is able to drive the transcription of downstream sequences, thus behaving as a promoter (Figure 6A). As a member of the *Tc1-mariner* superfamily, *Bari3* has a weak promoter, ensuring low transposase levels. In fact, high transposase activity would probably be deleterious for the host genome, or would trigger inhibitory mechanisms to block transposition (for example, overexpression inhibition). The presence of a promoter in the analyzed sequence suggests that the transcription of the transposase gene is a possible event *in vivo*, further supporting the hypothesis that *Bari3* is an active element.

The presence of a functional Bari3 transposase was tested both by *in silico* and molecular approaches. Nuclear localization of the transposase is essential for the mobilization of chromosomal copies of the transposon. Here, we used a deletion approach and found that a NLS motif is present within the first 168 amino acids of *Bari3* transposase (Figure 3). We mapped this domain in position 103 to 121 of the transposase’s primary sequence, based on comparative analysis of 14 transposases encoded by transposons of the *Tc1-mariner* superfamily (Figure 4). Furthermore, by combining multiple alignment and protein motif detection analysis, we present clear evidence that the transposase present in *Bari3* possesses all typical domains of the *Tc1-mariner* transposases (Figure 3), including a correctly spaced DDE amino acidic triad involved in the catalysis. Similar analysis suggested that *Bari3* transposase contains a N-terminal DNA binding domain, and this finding was further investigated by a new experimental strategy presented in this paper (see below).

The transposon-transposase interaction is also a necessary condition for the transposition reaction to occur. Taking advantage of the dual properties of the left terminal sequence of TIR-containing transposons (that is, to act as a promoter and as binding site for the transposase), we described a new approach based on a modified promoter luciferase assay and demonstrated the transposase-left TIR interaction. This assay is based on the assumption that if the transposase/left TIR interaction occurs, then a reduction of the reporter activity (that is, luciferase) should be observed (Figure 6 B). Indeed, the presence of *Bari3* transposase resulted in a significant reduction of the reporter activity, suggesting the presence of transposase binding sites within the left TIR. The left TIRs of *Bari3* and *Bari1* present 62% of sequence similarity (RC and RMM unpublished observation), and share also share three highly conserved stretches of DNA in the transposon termini [14]. In *Bari1,* these stretches represent the transposase binding sites [24], and their high similarity strongly suggests that these sequences are also genuine binding sites for the *Bari3* transposase.

### The possible role of transposase-processed transcripts in *Bari3* regulation

Nothing is currently known about the regulation of *Bari3* in *D. mojavensis*, but it is likely that it must be subjected to regulatory mechanisms that contain its transposition.

A number of transposition repressive mechanisms, regulating *Tc1-mariner* elements, have been discovered to date, starting from self-regulation (overexpression inhibition [47-49], post-translational modifications of the transposase [50,51], self-encoded repressors [52,53]) to the cell-developed control systems (siRNA [54] and piRNA [55] pathways, chromatin-level transcriptional repression [56]), or simply stochastic accumulation of detrimental mutations in the transposase-coding gene [57]. Some of these control mechanisms have been demonstrated for *Bari*-like elements [58-60]. In light of our results, similar controlling mechanisms can be hypothesized for *Bari3*.

Similarly to other transposons, including its closest relative *Bari1*, epigenetic regulation of *Bari3* mediated by piRNA could be expected due to the presence of small RNA in the genome of *D. mojavensis* (generated in unidentified genomic loci). Furthermore, the integrity of the left and right TIRs suggests that both could drive transcription, which might result in the formation of dsRNA molecules able to trigger the siRNA/piRNA response.

In addition, our observation that the transposase gene transcripts may undergo processing could be also taken in consideration in future studies concerning additional regulation mechanism controlling *Bari3*.

We have recently reported that the *Bari1* element is subjected to transcript processing when the transposase is overexpressed in cultured cells or *in vivo* in an unrepressed genetic background due to mutations in key genes controlling the piRNA pathway [24]. Here we have investigated the possibility that *Bari3* transcripts could have similar post-transcriptional processing in similar experimental conditions. *In vitro* analyses performed by transient overexpression of the plasmid pAC/ASE3 in S2R + cells revealed the presence of a cDNA of unexpected size, which is the result of a canonical splicing process and potentially encodes for a transposase lacking the binding domain (Figure 5 and see Additional file 3).

Interestingly, a processed transcript sharing the same structural features has been identified also after overexpression in *Drosophila* embryos. It is worth noting that embryos differentially process the *Bari3* transposase transcript in the anterior pole or in the posterior pole, suggesting that the transposase RNA processing is probably soma-specific or it relies on the presence of splicing factors not uniformly distributed along the longitudinal axis of the embryo.

The finding that embryos process transposase transcripts in the anterior pole is slightly surprising as processing would be more likely to occur in the posterior pole of the embryo where the germ line is going to be developed. The somatic post-transcriptional control is somehow reminiscent of the somatic splicing of *P-element* in *D. melanogaster*[61]. We cannot hypothesize obvious functions for the protein encoded by the processed *Bari3* transcript, which is formally a N-terminal truncated version of the wild type *Bari3* transposase, thus lacking the DNA binding function and part of the catalytic domain [see Additional file 3].

The presence of splicing sites in transposase encoding genes has been reported for other well-studied transposons like the *Ac* element [62], whereas *Tc1-mariner* elements do not usually contain introns in their transposase coding genes. However cryptic splicing sites can be activated following transposon insertions within the host genes’ coding regions [63,64], a process that allows genes to acquire novel exons and to evolve new splicing and expression patterns [65].

Our results demonstrated the presence of cryptic splice sites in *Bari3*, probably activated upon overexpression in cultured cells and in *D. melanogaster* embryos. It is possible that activation of these sites could constitute an additional, or an alternative, method of protection against transposition. The hypothetical protein product encoded by the detected spliced transcript should have lost the DNA binding activity, the protein-protein interaction domain, the NLS and part of the catalytic domain (see Figure 5 and Additional file 3), and consequently, it should not negatively influence transposition efficiency. By contrast, the partial depletion of the full-length transposase-encoding mRNAs, resulting from its splicing, could have an impact on *Bari3* transposition, due to the lower transposase mRNA amount that can be translated.

Interestingly, in a recent paper the splicing process has been linked to the siRNA pathway in the regulation of transposons in the encapsulated yeast *Cryptococcus neoformans*. The presence of suboptimal splice sites in transposons’ transcripts could lead to stalling of the spliceosome, which produces partial or incomplete mRNA precursors and consequent triggering of the siRNA/piRNA response [66]. It can be speculated that similar mechanisms could be involved in the control of *Bari3* transposition.

## Conclusions

The characterization of the *Bari3* transposon presented in this paper increases the current knowledge on the *Tc1-*like elements. Our results justify further studies on the *Bari* family of transposons. These elements are intriguing both for their widespread diffusion in Drosophilids and for their structural diversity. To fully understand the biology of these TEs, it will be necessary to undertake studies connecting structural (for example, short versus long TIRs) to functional features of different *Bari* subfamilies (namely *Bari1* and *Bari3*). In this context, a remarkable result is the transcript processing, which appears as a recurrent feature in active elements of the *Bari* family [24]. A probable scenario could be the existence of a pathway leading to the depletion of the mRNA transposase source in response to a defined threshold, blocking transposition upon failure of other control mechanisms.

## Methods

### *Drosophila* stocks and cell culture maintenance

*Drosophila mojavensis* stocks were obtained from the Drosophila Species Stock Center (University of California, San Diego) and reared on banana/*Opuntia* medium. Fly stocks from different species were maintained on standard cornmeal-agar medium at 24°C.

S2R^+^ cells (Drosophila Genomics Resource Center, Bloomington, USA) were cultured in Schneider’s insect medium supplemented with 10% FBS, 1% penicillin/streptomycin, at 26°C. HeLa and HepG2 cells were grown in Dulbecco’s Minimum Essential Medium supplemented with 10% FBS, 200 mM glutamine, 1% penicillin/streptomycin, and maintained at 37°C with 5% CO_2_.

### Plasmid construction

Standard cloning procedures were used to obtain the plasmids used in this study [67]. A list of the oligonucleotides used in PCR steps is provided as additional file [see Additional file 4]. The full length *Bari3* element (pT/moja11) was PCR-isolated from *D. mojavensis* DNA using the FL2_for/FL2_rev primers targeting the element in the *D. mojavensis* scaffold_6540 and was cloned into the pGEM-T easy vector (Promega, Madison, WI, USA).

Bari3_UP/Bari3_Low, Bari3_UP/Bari3_N-Ter Low, and Bari3_C-Ter Up/Bari3_Low were used to amplify and were subsequently cloned into the KpnI and NotI sites of pAC5.1/V5-His vector (Invitrogen, Carlsbad, CA, USA), DNA sequences encoding respectively the full length *Bari3* transposase gene (pAC/ASE3), the first 168 (pAC/Δ169-339) or the last 171 amino acids of the transposase (pAC/Δ1-168). The fusion constructs were subcloned in pcDNA3.1 (Invitrogen, Carlsbad, CA, USA) using EcoRI and BamHI restriction sites, obtaining the plasmids pcDNA/ASE3, pcDNA/Δ169-339, and pcDNA/Δ1-168. The plasmid, pcDNA/ASE1 has been described in [24].

The Ba3TIR was amplified from the pT/moja11 plasmid with the TERBa3_UP/TERBa3_LOW primers and cloned into the XhoI and NcoI sites of the pGL3B vector (Promega, Madison, WI, USA) to obtain the pGL3B-Ba3LTIR plasmid.

The Ba1TIR was amplified from the p28/47D [43] plasmid with the TERBa1_UP/TERBa1_LOW primers and cloned into the XhoI and NcoI sites of the pGL3B vector (Promega, Madison, WI, USA) to obtain the pGL3B-Ba1LTIR plasmid.

The *copia* promoter was amplified from the pCoBLAST vector (Promega, Madison, WI, USA) with the copia_for/copia_rev primers and cloned into the XhoI and NcoI sites of the pGL3B vector to obtain the pGL3B-copia plasmid.

pcDNA3.1**/**myc-His(−)/lacZ (Life Technologies, Grand Island, NY, USA) was used to express β-Galactosidase.

All plasmids were sequence-verified.

### DNA extraction, Southern blotting and fluorescence *in situ* hybridization

Genomic DNA was prepared according to [68]. DNA samples were digested with the EcoRI restriction enzyme (New England Biolabs Inc, Ipswich, MA, USA), which cuts once in the reference sequence of *Bari3* (see Figure 2A), electrophoresed, blotted onto Hybond N filters and hybridized under high stringency hybridization conditions [67]. Probes used in Southern blot hybridization were labeled with [α-32P] dATP by random priming.

Polytene chromosomes were prepared from salivary glands of third instar *D. mojavensis* larvae essentially as described in [69]. Probes used in fluorescence *in situ* hybridization were labeled by nick-translation with the Cy3-dCTP fluorescent precursor (GE Healthcare Life Sciences, Pittsburgh, PA, USA), and chromosomes were counterstained with 4,6-diamidino-2-phenylindole-dihydrochloride (DAPI). Finally, digital images were obtained using an Olympus epifluorescence microscope equipped with a cooled CCD camera. Gray scale images, obtained separately for Cy3 and DAPI fluorescence using specific filters, were pseudo-colored and merged to produce the final image using Adobe Photoshop.

A 592-bp probe was amplified from the pT/moja11 clone using primers Moj11_534Up/Moj11_1126Low, and used for all hybridization experiments.

### Embryo microinjection and post-injection care

Microinjection of pre-blastoderm embryos was performed essentially as described in [70] with little modifications. Females of the *Oregon-R* strain were allowed to lay eggs for one hour on grape juice agar plates. Eggs were washed with a 70% ethanol (v/v) solution, and aligned manually on a coverslip, mounted on a microscope slide, briefly desiccated, covered with halocarbon oil and injected at either their posterior or anterior pole with a capillary needle attached to an Eppendorf Femtojet microinjector. Needles for microinjection were obtained from borosilicate glass capillaries, pulled with a Narishige PC-10 puller. Concentration of injected DNA was usually 0.5 to 0.8 mg/ml. After injection, the cover slip containing the embryos were carefully removed from the slides and transferred to grape juice plates. After incubation at 18°C for 24 hours, embryos were further subjected to RNA extraction.

### Plasmid transfection and immuno-detection of recombinant proteins

One day prior to transfection cells were seeded and let grow into 6-well plates containing sterile glass coverslips. Respectively 1 × 10^6^ and 5 × 10^5^ S2R^+^ and HepG2 cells were transfected with 1 μg of purified plasmids DNA using TransIt LT1 (Mirus Bio, Madison, WI, USA).

For immunofluorescence staining, the cells attached to slides were washed with phosphate-buffered saline and fixed with 4% formaldehyde for 10 minutes at room temperature followed by three washes in PBS. Blocking was performed with a solution containing 10% fetal bovine serum and 0.5% of Triton X-100 for 30 minutes followed by two washes in PBS for 2 minutes each.

Cells were incubated with a dilution 1:500 of V5 antibody (Invitrogen, Carlsbad, CA, USA) conjugated with fluorescein isothiocyanate (FITC) fluorochrome for 2 hours. After three washes in PBS, the cells were stained with DAPI (4',6-diamidino-2-phenylindole) and mounted with anti-fade 1,4-diazabicyclo[2.2.2]octane (DABCO).

Slides were imaged under an Olympus (Tokyo, Japan) epifluorescence microscope equipped with a cooled CCD camera. At least 100 positive cells per slide were observed. Grey-scale images, obtained by separately recording FITC and DAPI fluorescence, were pseudo-colored and merged to obtain the final image using Adobe Photoshop program.

### Promoter luciferase assay

S2R + cells were transfected with 1 μg of the appropriate plasmid (either pGL3B-Ba1LTIR, pGL3B-Ba3LTIR pGL3B-copia or the empty pGL3B). *Renilla* luciferase construct (pRL-SV40; Promega, Madison, WI, USA) was used for normalization. Luciferase expression was measured by the detection of luminescence using the dual luciferase reporter assay system (Promega, Madison, WI, USA) according to the manufacturer instructions. Measurements were recorded on GLOMAX 20/20 luminometer (Promega, Madison, WI, USA). The average expression level from three replicate transfections was normalized to the *Renilla* luciferase co-transfection control. This value was further normalized to the average expression level from three normalized replicates of the pGL3B-copia plasmid to yield a relative luciferase activity estimate.

For the luciferase activity suppression assay HeLa cells were previously transfected with plasmid expressing either transposase (pcDNA/ASE3, pcDNA/ASE1) or β-Galactosidase (pcDNA3.1**/**myc-His(−)/lacZ) (Invitrogen, Carlsbad, CA, USA).

Error bars represent the standard deviation. Student’s t test was used to evaluate statistical significance.

### Transcriptional analysis

RNA was extracted with TRIzol® Reagent (Invitrogen, Carlsbad, CA, USA). Cultured cells were directly processed after two washes in PBS 1X. Quantitation and estimation of RNA purity were performed using a NanoDrop spectrophotometer.

A total of 1 μg RNA was converted to cDNA using the QIAQuick reverse transcription kit (Qiagen, Hilden, Germany) and following the manufacturer’s instruction. cDNA samples from transfected S2R + cells and from injected embryos were amplified with the AC5_forward/BGH_Rev primers. Nested PCR was performed using the Bari3_Up1/V5_rev primers.

### *In silico* methods

Pairwise alignments were performed using either the NCBI online tools or the LALIGN tool (http://embnet.vital-it.ch/software/LALIGN_form.html).

Multiple alignments were performed using the Multalin tool (http://multalin.toulouse.inra.fr/) [34]. Protein secondary structures predictions were performed using the PhD secondary structure prediction method (https://www.predictprotein.org/) [71]. Sequences used for construction of the multiple alignment in Figure 4 were retrieved from the Repbase database (http://www.girinst.org) [72].

## Abbreviations

CCD: charge-coupled device; CHIP: chromatin immunoprecipitation; DAPI: 4',6-diamidino-2-phenylindole; DR: direct repeat; EMSA: electrophoretic mobility shift assay; FITC: fluorescein isothiocyanate; HTH: helix-turn-helix; IR-DR: inverted repeat-direct repeat; Kbp: kilobase pairs; NLS: nuclear localization signal; ORF: open reading frame; PBS: phosphate-buffered saline; PCR: polymerase chain reaction; TE: transposable element; TIR: terminal inverted repeat.

## Competing interests

AP, RC, RMM have applied for a patent related to part of the content of this manuscript. The remaining authors declare that they have no competing interests.

## Authors’ contributions

AP, RM, and RMM, performed the experiments. RC and RMM conceived the study, participated in its design and coordination, and drafted the manuscript. All authors read and approved the final manuscript.

## Supplementary Material

Additional file 1 **Sequence and main features of *Bari3.***
Click here for file

Additional file 2 ***Bari3 *** **in the reference genome of *Drosophila mojavensis.***
Click here for file

Additional file 3 **Structure of the spliced *Bari3 *** **transcript and its encoded protein.** Click here for file

Additional file 4 List of the primers used in this work. Click here for file
