# Supplementary material for: The Drosophila mojavensis Bari3 transposon: distribution and functional characterization
Source: Mob DNA. 2014 Jul 8;5:21. doi: 10.1186/1759-8753-5-21 (PMC4120734; doi:10.1186/1759-8753-5-21)
Supplement: Additional file 1 — Sequence and main features of Bari3. [file 1759-8753-5-21-S1.docx]

**TAcagaggtggtcaaaagtaattacacaccgagcttttttctgcatgctcagctttaaaagcttgttgttgttgttgttgttcgctgtttttgaaaaaaagcttttgtgtcataaagcttattctgtgtagaagcgatcggcaaaaagaaattgctaaaataacggtacaggttcgtagatatagcaaaaagaacagttacctagaggttgtcaaaagtatttacacacattttttttgcgtcataagtatttacaca**ATGTAAATTTGCCTTTAGTCTTTGATTTGCGTTGATCATGTGCGGTCTTTGACTAAGATCGGAATTCAATTTGTAGTTGAAATTGTAGATGTGCTGTAAAatggtaaaaacaaaggaactatcagtcgaaacaagatccgagatcgtaaccaagtttaagagtggtgttggggcatcggagttggtaaagatattcaaaatatctagaaaaacggtgtataacttggttaacaaaagggatacacttggaaatttgaataatttacaccgaagtgggcgaaagccagcattgaacaccagggaatgcagaagacttgtcagcacattcgtagaaaaccccgagataagcccagttaaagtgtctgcagcatcacaacagataattggtaaacatgtcagtgatgctactattcgtagaaggttaaaggatgttgacataaacacctatgtcgtccgaaaaatcattgatatctcggaagcaaacaaagcaaaacgtcttgcttttgctttagagtacattgaaaagcctttggagttttggtacaacattttgtggacagatgaggttgcatttcagtttcaaggctcatttcgaaagacttttatgcatttaagtcgacaaaataaaaaaactgcagtgcagcctcttagcaggtttggaggtggcacagtaatgttttggggttgcatgtcgtactatggttttggagattttgtgccaatagagggcactctgacccaatcacgatatttgcacatattaaatgacaatgcttttacttcggcgaacaaactatttccaacaatcgaatgggtattgcagcaagacaatgctccatgccacaaaggctcaatacccaccaagtttttggcggacttagatcaagcaatcctgccctggcctgcgcaaagtccggaccttaacattattgaaaatgtttgggctttaataaaatccaaacggctattcaaaaaggaccggaaacgggatgataccattcaggaagttaccggaatatggtcggagctcacagttgacttcgcccgatcattggtaacatcaattccagccaggcttcaggctgtaattgacgaaaaagggggaatgacaaaatactaaATTAAATAAATAAACCCTTTATTATGTTCAACAAAAATTAAAAATAAATAACATTAATTAATGCCATTAAAAAATGTTTAAATTTT**gtgtaaatacttatgacgcaaaaaaaatgtgtgtaaatacttttgacaacctctaggtaactgttctttttgctatatctacgaacctgtaccgttattttagcaatttctttttgccgatcgcttctacacagaataagctttatgacacaaaagctttttttcaaaaacagcgaacaacaacaacaacaacaagcttttaaagctgagcatgcagaaaaaagctcggtgtgtaattacttttgaccacctctgTA**

**Figure legend**

**TA** duplicated target site

**terminal inverted repeats (TIRs)**

**Direct Repeats**

SPACER

**Transposase gene**

gt, ag donor and acceptor of splicing detected in this study.

**
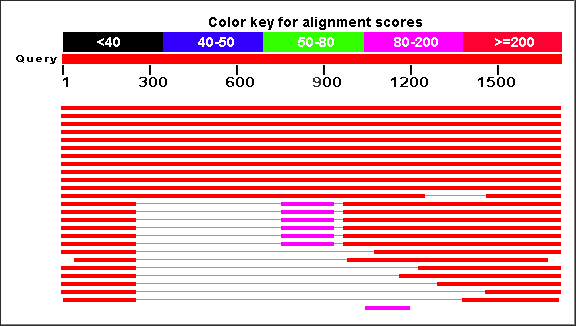
**
