# Supplementary material for: The Drosophila mojavensis Bari3 transposon: distribution and functional characterization
Source: Mob DNA. 2014 Jul 8;5:21. doi: 10.1186/1759-8753-5-21 (PMC4120734; doi:10.1186/1759-8753-5-21)
Supplement: Additional file 2 — Bari3 in the reference genome of Drosophila mojavensis. [file 1759-8753-5-21-S2.docx]

A


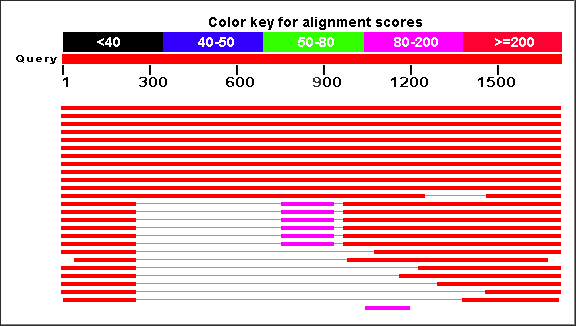


B

**SUBSET A**) Genomic sequences containing full-length Bari3 insertions in D. mojavensis.

AAPU01011127.1

AAPU01011020.1

AAPU01010985.1

AAPU01010715.1

AAPU01010530.1

AAPU01008278.1

AAPU01010214.1

AAPU01009778.1

AAPU01010557.1

AAPU01011156.1

AAPU01009527.1

**SUBSET B**) Genomic sequences containing degenerated Bari3 insertions in D. mojavensis.

AAPU01000425.1*

AAPU01010640.1

AAPU01010045.1

AAPU01010721.1

AAPU01009629.1

AAPU01010841.1

AAPU01010708.1

AAPU01000483.1

AAPU01009880.1

AAPU01011692.1

AAPU01006172.1

AAPU01007307.1

AAPU01011230.1

AAPU01011664.1

AAPU01009831.1

*Truncated Bari3 element due to limited size of the contig
