# Supplementary figures and images for: The Drosophila mojavensis Bari3 transposon: distribution and functional characterization
Source: Mob DNA. 2014 Jul 8;5:21. doi: 10.1186/1759-8753-5-21 (PMC4120734; doi:10.1186/1759-8753-5-21)

## Slide 1
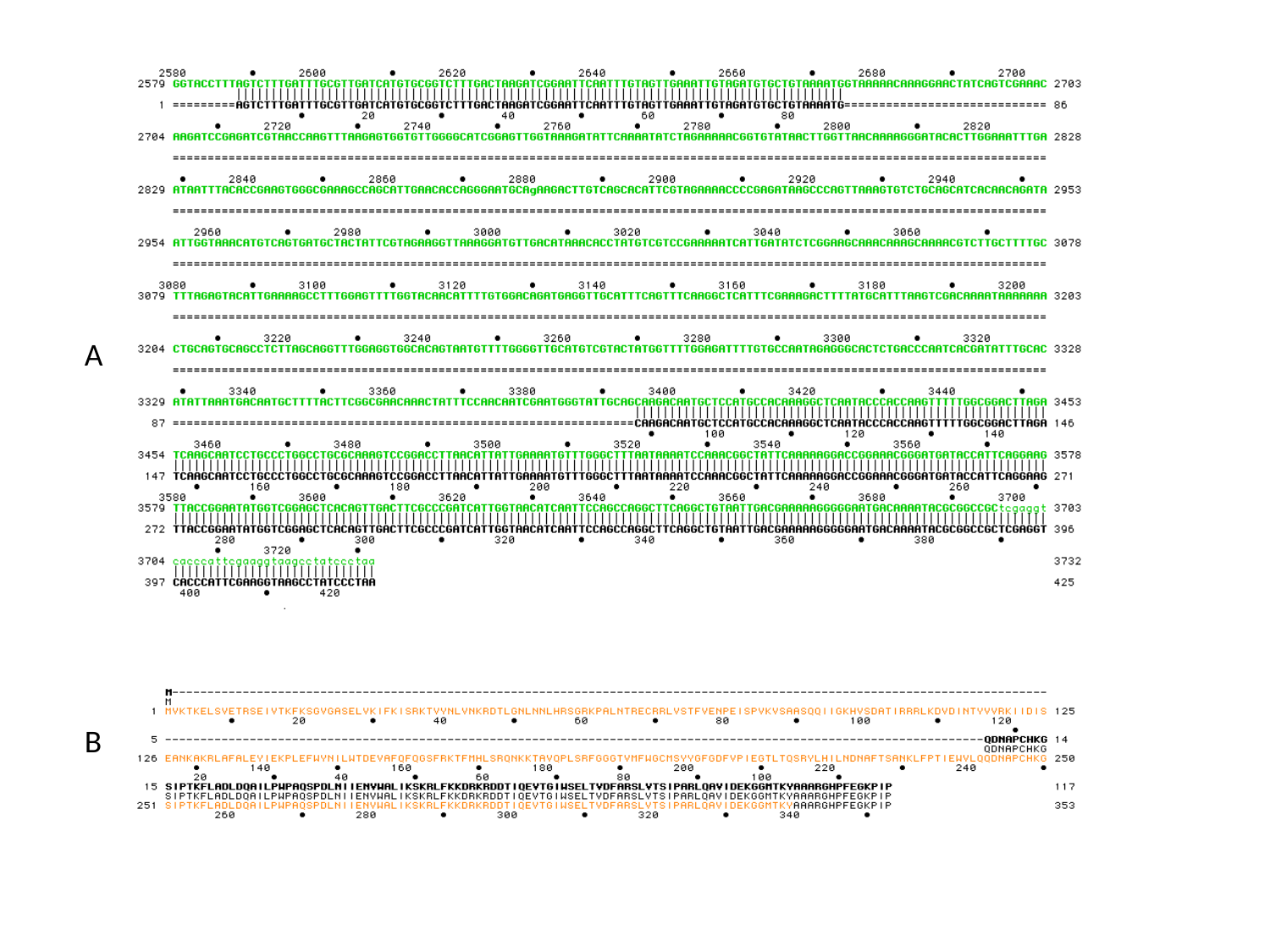

A
B

Supplement: Additional file 3 — Structure of the spliced Bari3 transcript and its encoded protein. [file 1759-8753-5-21-S3.pptx]
