# Supplementary material for: The Drosophila mojavensis Bari3 transposon: distribution and functional characterization
Source: Mob DNA. 2014 Jul 8;5:21. doi: 10.1186/1759-8753-5-21 (PMC4120734; doi:10.1186/1759-8753-5-21)
Supplement: Additional file 4 — List of the primers used in this work. [file 1759-8753-5-21-S4.docx]

| **Oligo Name** | **Sequence** |
| --- | --- |
| Bari3_C-Ter UP | gataggtaccatgcatttaagtcgacaaaata |
| Bari3_N-Ter Low | gatagcggccgcaaaagtctttcgaaatga |
| Bari3_Up1 | gataggtacctttagtctttgatttgcgtt |
| Bari3_Low1 | gatagcggccgcgtattttgtcattccc |
| TERBari3_up | gatactcgagcagaggtggtcaaaagtaa |
| TERBari3_low | gataccatggtttacagcacatctacaatt |
| BGH Reverse | tagaaggcacagtcgagg |
| V5 Reverse | accgaggagagggttagggat |
| Ac5 Forward | acacaaagccgctccatcag |
| Copia_For | gatactcgagatctaggattgggaacccctcatcat |
| Copia_Rev | gataccatggcttcgcataaagggaacct |
| FL2_for | caatcgtgcaaaactgact |
| FL2_rev | tccgtgacaagacaaaattc |
| TERBari1_up | gatactcgagcagtcatggtcaaaatta |
| TERBari1_low | gataccatggtttatttaataactgtaa |
| Moj11_534Up | cgaaagccagcattgaacac |
| Moj11_1106Low | aaaacttggtgggtattgag |

**Supplemental table. List of oligonucleotide used in this study.**
